# Primary transcript enrichment reveals a density-dependent *Wolbachia* transcriptome and active mosquito RNA viruses

**DOI:** 10.3389/fmicb.2026.1860204

**Published:** 2026-08-12

**Authors:** Amit Sinha, Clotilde K. S. Carlow, Zhiru Li

**Affiliations:** New England Biolabs, Inc., Ipswich, MA, United States

**Keywords:** arboviruses, Cappable-seq, metavirome, primary transcripts, transcriptomics, type IV secretion system, *Wolbachia w*AlbB

## Abstract

*Wolbachia pipientis* is a widespread obligate intracellular bacterium and an effective biocontrol agent being deployed in the field against mosquito-borne viral diseases. Investigating the transcriptomic dynamics of *Wolbachia* within host cells is highly desired but is fundamentally challenged by the overwhelming abundance of eukaryotic host RNA and bacterial ribosomal RNA. Here, we demonstrate that Cappable-seq specifically captures the active, primary transcriptome of *Wolbachia* while drastically reducing host background. We applied this methodology to monitor *Wolbachia* gene expression changes over a single host cell passage cycle, tracking the transition from early infection to a high-titer maintenance phase. Differential gene expression analysis identified the upregulation of *rpoH* transcription factor, stress-response chaperones and WO prophage elements. This was coupled with a continuous downregulation of key host-manipulation effectors, including Ankyrin-repeat domain-containing proteins and secretion systems, alongside the broad suppression of metabolic transporters over the time-course. In addition, we discovered that Cappable-seq concurrently co-enriches the transcripts and genomes from various single-stranded RNA viruses that are known to be present in these mosquito cells. In summary, Cappable-seq technology provides a robust framework to investigate complex, tripartite host-microbe-virus interactions, demonstrating that *Wolbachia* actively senses and adapts to the dynamic constraints of its intracellular environment by regulating its transcriptome.

## 1 Introduction

*Wolbachia pipientis* is a maternally inherited obligate intracellular endosymbiont of arthropods (Werren et al., 2008) and filarial worms (Landmann, 2019). Its widespread distribution among insects makes *Wolbachia* a great model for exploring biological interactions between endosymbionts and their invertebrate host cells, as well as the diverse phenotypes it induces. While significant efforts have focused on understanding *Wolbachia*-induced host phenotypes, knowledge of the molecular mechanisms underlying the intracellular lifestyle of *Wolbachia* remain limited.

All *Wolbachia* possess an evolutionary conserved type IV secretion system (Pichon et al., 2009; Li and Carlow, 2012) that *Wolbachia* use to secrete effectors, often characterized by eukaryotic-like domains (Rice et al., 2017; Carpinone et al., 2018), to establish and sustain their intracellular niche and lifestyle. While the T4SS genes, which cluster in two loci, were previously found to be transcriptionally active (Rancès et al., 2008), the transcription dynamics accompany the invasion and maintenance mechanisms during an active cellular infection cycle remain difficult to resolve.

Transcriptomic profiling of obligate intracellular bacteria and viruses is a powerful tool for understanding host-microbe interactions, pathogenesis, and intracellular survival. However, transcriptomic studies of bacterial endosymbionts that resides within their eukaryotic hosts’ cells, remains challenging. In typical infected host cell models, bacterial messenger RNA (mRNA) often constitutes a minuscule fraction of the total RNA pool, frequently falling below 1%. Consequently, achieving sufficient sequencing depth using standard RNA-seq to accurately capture dynamic bacterial gene expression directly requires deeper sequencing depth, making comprehensive temporal analyses difficult and costly. One strategy to overcome this problem is the isolation and purification of endosymbiont cells, but this suffers from low bacterial recovery and potential artifacts introduced during the purification process. Another approach to overcome this challenge is ribosomal RNA (rRNA) depletion, which relies on hybridization to a panel of probes specific to the rRNA genes of both the host and the symbiont, followed by RNase H digestion. However, this approach presents significant limitations. Custom rRNA probes must be designed and optimized for every new host-symbiont pairing (Cantin et al., 2024), which can be a time-consuming and costly process. Conversely, relying on pre-existing generic probes often yields suboptimal depletion due to sequence divergence from the model organisms. Furthermore, even when this technique successfully removes abundant rRNAs, a second depletion of polyadenylated eukaryotic mRNA is usually necessary to remove the massive eukaryotic host mRNA background and improve coverage of bacterial transcripts. Yet another alternative approach utilizes a 5′-phosphate-dependent exonuclease such as TEX (Sharma et al., 2010), to degrade processed RNA molecules containing a 5′- monophosphate, including rRNAs. However, variations in the efficiencies of the enzymatic degradation and the subsequent bacterial mRNA enrichment have been observed (He et al., 2010).

To reduce the background from rRNA and host RNA, strategies that positively select for bacterial transcripts are highly advantageous. A method named Cappable-seq exploits the unique 5′-triphosphate native to primary prokaryotic transcripts, allowing for their enzymatic capping with a biotinylated GTP and subsequent physical enrichment using streptavidin bead-based pulldown (Ettwiller et al., 2016). This enables the separation of primary bacterial transcripts from both mature rRNAs (which lose their 5′-triphosphate during nucleolytic processing), and eukaryotic mRNAs (which are naturally 5′-capped). This method has been successfully applied to study *Mycobacterium* under hypoxic stress condition (Huang et al., 2021). It also enabled the enrichment (3.8–4.8-fold) of desired *Wolbachia* mRNA transcripts from total RNA samples of *Brugia malayi* (Luck et al., 2017). Recently, this approach was utilized to annotate the transcriptional start sites of *Wolbachia* to single nucleotide resolution and to study the differential regulation of *Wolbachia w*Mel and *w*AlbB transcriptomes during heat and antibiotic stressors (Suliman et al., 2025).

Cell lines containing *Wolbachia* represent a suitable model to explore the symbiotic relationship and have been used extensively in molecular, biochemical, and genetic studies (Voronin et al., 2012; Saucereau et al., 2017). The Aa23 cell line, derived from *Wolbachia*-infected *Aedes albopictus* mosquito embryos and containing *Wolbachia* strain *w*AlbB, was one of the first such cell lines developed (Sinkins et al., 1995; O’Neill et al., 1997) and has a complete genome sequence available (Sinha et al., 2019). While *w*AlbB is dependent on the host cell to grow and replicate, studies also found that purified *Wolbachia* can remain viable in an extracellular environment and infect mosquito cell lines, ovaries, and testes when co-cultured (Rasgon et al., 2006; Hughes et al., 2012). Furthermore, evidence indicates that *Wolbachia* are capable of exiting the host cell to undergo horizontal cell-to-cell transfer, even between divergent hosts (White et al., 2017). As the host cells grow throughout a standard cell passage, *Wolbachia* needs to rapidly adapt its expression profile to transition from establishing its niche to maintaining a stable, density-dependent intracellular state, balancing a dynamic equilibrium with its host cells.

In this study, we establish an optimized Cappable-seq protocol as a highly effective method for resolving the transcriptomes of endosymbionts within infected mosquito Aa23 cells. By comparing Cappable-seq libraries to standard total RNA and XRN-1 exonuclease-treated controls, we demonstrate the robust, selective enrichment of *Wolbachia* primary transcripts. Furthermore, we report that Cappable-seq also efficiently co-enriches the active, uncapped viral community (including San Gabriel mononegavirus and Tombus-like viruses). Applying this targeted methodology, we mapped the active transcriptome of the *Wolbachia* strain *w*AlbB over a single passage of mosquito host cell. This high-resolution temporal profiling identified distinct transcriptional shifts as the bacterial population expanded, revealing a co-ordinated upregulation of energy metabolism and a downregulation of T4SS structural components and host-modulating effectors following successful host-cell establishment.

## 2 Materials and methods

### 2.1 Mosquito cell culture and density experiments

The *A. albopictus* Aa23 cell line infected with the *Wolbachia* strain *w*AlbB was maintained at 28 °C in T75 tissue culture flasks. Cells were cultured in an insect medium comprising 45% Mitsuhashi and Maramorosch medium (HiMedia), 45% Schneider’s *Drosophila* medium (Sigma-Aldrich), 10% fetal calf serum (FCS), and 0.18% L-glutamine. Cultures were passaged every 4–7 days upon reaching confluency at a 1:4 split ratio (3 mL of suspended cells added to 12 mL of fresh medium).

For the single-passage host cell growth experiments, confluent cells (designated as Day 0) were detached using a cell lifter and resuspended in fresh medium to a concentration of 6 × 10^5^ cells/mL. Five milliliters of this suspension were seeded into individual T25 flasks. Cells were subsequently harvested after 3, 4, 5, 6, and 7 days of incubation for downstream DNA and RNA extraction.

### 2.2 DNA and RNA extraction

Genomic DNA was isolated using the Monarch Spin gDNA Extraction Kit (New England Biolabs, #T3010) according to the manufacturer’s instructions. Total RNA was extracted utilizing a hybrid QIAzol-chloroform extraction method followed by column purification. Briefly, cell pellets generated from 1 to 2 mL of culture suspension were lysed in 1 mL of QIAzol Lysis Reagent and incubated at room temperature for 3 min. The QIAzol-sample mixture was either processed immediately or stored at −80 °C.

For phase separation, 200 μL of 100% chloroform was added per 1 mL of QIAzol lysate. The mixture was thoroughly homogenized, incubated at room temperature for 3 min, and centrifuged at 12,000 × *g* for 15 min at 4 °C. Following centrifugation, 500 μL of the upper aqueous phase was carefully transferred to a DNA LoBind tube (Eppendorf), to which 750 μL of absolute ethanol was added. The resulting mixture was then purified utilizing the Monarch Total RNA Miniprep Kit (New England Biolabs, #T2110S), which included an on-column DNase treatment as specified by the manufacturer’s protocol.

### 2.3 Quantification of relative *Wolbachia* density

To quantify the relative intracellular density of *Wolbachia* within the Aa23 cell line across the temporal passage, total genomic DNA was extracted from three independent biological replicates per time point. TaqMan multiplex quantitative PCR (qPCR) was performed using the Luna Universal Probe qPCR Master Mix (New England Biolabs, M3004) alongside specific primers and probes targeting the bacterial *Wolbachia* 16S rRNA gene and the eukaryotic host *A. albopictus* 18S rRNA gene (Table 1). Relative quantification was performed utilizing the relative standard curve method. Briefly, a standard curve was generated for both targets within the same run using a series of of 7 consecutive 10-fold serial dilutions of a highly concentrated, pooled Aa23_*w*AlbB genomic DNA reference sample (Supplementary Figure 1). The amplification efficiencies for the 16S and 18S standard curves were 98.9% and 100.2%, respectively, with correlation coefficients (R^2^) of 0.999 for both targets. For each experimental sample, the relative starting DNA quantities for the 16S and 18S targets were interpolated from their respective standard curves based on their quantification cycle (Cq) values. To normalize for variations in total DNA input and host cell number, the relative *Wolbachia* titer was calculated as the ratio of the 16S target quantity to the 18S target quantity. All samples were run in technical triplicates, and the reported relative density values represent the mean of the three independent biological replicates.

**TABLE 1:** Primer sequences targeting 16S gene of *Wolbachia* and 18S gene of *Aedes albopictus*.

qPCR primers	Sequence 5′ to 3′
*Wol16S*_qPCRF	CACAGAAGAAGTCCTGGCTAAC
*Wol16S*_qPCRprobeFAM	/56-FAM/CGGTAATAC/ZEN/GGAG AGGGCTAGCGTTA/3IABkFQ/
*Wol16S*_qPCRR	CGCCCTTTACGCCCAATAA
*Ae18S*_qPCRF	CCGTGATGCCCTTAGATGTT
*Ae18S*_qPCRprobe 5′HEX	/5HEX/AATGT GCGC/ZEN/AGCAACGCGTATC /3IABkFQ/
*Ae18S*_qPCRR	ATGCGCATTTAAGCGATTTC

### 2.4 Cappable-seq primary transcript enrichment

Primary bacterial transcripts were captured using the modified Cappable-seq method. For the capping reaction, 5–10 μg of DNase-treated RNA was combined with 5 μL of 10X Vaccinia Capping Enzyme Buffer (NEB #M2080), 5 μL of 5 mM 3’-desthiobiotin-guanosine triphosphate (DTB-GTP) (NEB #N0761), 5 μL of Vaccinia Capping Enzyme (NEB #M2080), 5 μL of Inorganic Pyrophosphatase (NEB #M2403), 1 μL of RNase Inhibitor (NEB #M0314), and nuclease-free water (NEB #B1500) to reach a final reaction volume of 50 μL. The mixture was incubated at 37 °C for 1 h. The capped RNA was subsequently purified utilizing the Monarch RNA Cleanup Kit (NEB #T2030S), incorporating five consecutive washing steps to ensure the complete removal of free, unincorporated residual DTB-GTP.

To isolate the capped primary transcripts, the purified RNA was subjected to one or two consecutive rounds of streptavidin bead enrichment. Streptavidin Magnetic Beads (NEB #S1421S) were initially prepared by washing twice in a low-salt buffer (10 mM Tris-HCl pH 7.5, 60 mM NaCl, 1 mM EDTA) and twice in a high-salt buffer (10 mM Tris-HCl pH 7.5, 300 mM NaCl, 1 mM EDTA), before being resuspended in the high-salt buffer to their original concentration of 4 mg/ml. For the primary binding step, 30 μL of the prepared streptavidin beads were combined with 30 μL of the DTB-GTP-capped RNA and incubated on a rotation mixer at room temperature for 30 min. The RNA-bead complexes were then washed three times with 200 μL of high-salt buffer and three times with 200 μL of low-salt buffer. The enriched RNA was eluted by performing two separate elutions with 11 μL of TE buffer (10 mM Tris-HCl pH 7.5, 1 mM EDTA) heated in a thermomixer at 80 °C and 600 rpm for 10 min. The combined ∼20 μL of eluted RNA was immediately subjected to a second identical round of streptavidin bead enrichment to maximize purity. Following the second enrichment, the bound RNA was eluted directly into the first-strand cDNA synthesis buffer containing random primers provided in the NEBNext Ultra II Directional RNA Library Prep Kit for Illumina (NEB #E7760) by incubating at 94 °C for 5 min to release and fragment the RNA for library preparation.

### 2.5 XRN-1 exonuclease treatment

To generate a control depleted of processed, 5′-monophosphate transcripts, total RNA was subjected to XRN-1 exonuclease digestion. The digestion was performed in a 50 μL reaction volume containing the total RNA, 5 μL of 10X NEBuffer 3, and 2.5 μL of XRN-1 (NEB #M0338). Following incubation at 37 °C for 1 h, the digested RNA was purified using the Monarch RNA Cleanup Kit (NEB #T2030S). The purified, XRN-1-treated RNA was subsequently utilized for library construction using the NEBNext Ultra II Directional RNA Library Prep Kit for Illumina (NEB #E7760), following the manufacturer’s instructions.

### 2.6 RNA-seq library preparation and sequencing

Sequencing libraries were constructed using the NEBNext Ultra II Directional RNA Library Prep Kit for Illumina (NEB #E7760) following the manufacturer’s instructions. For the Cappable-seq enriched RNA libraries, a 25-fold diluted adaptor was utilized, and the libraries were amplified using 13 PCR cycles. For the unenriched control libraries (starting with 100 ng of total RNA), a 5-fold diluted adaptor was used, and amplification was performed with 7 PCR cycles. Library quality and size distribution were assessed using an Agilent High Sensitivity D1000 ScreenTape system. The final libraries were pooled and sequenced on an Illumina NovaSeq 6000 platform using an SP flow cell with 2 × 150 bp paired-end chemistry. Raw sequence data have been deposited in the NCBI Sequence Read Archive (SRA) under the BioProject accession number PRJNA1392518.

### 2.7 Bioinformatics and data analysis

Quality control and pre-processing of the paired-end RNA-seq reads were performed using fastp v1.1.0 (Chen, 2025). To accurately distinguish endosymbiont reads from those of the host and its endogenous viruses, a combined reference genome was constructed utilizing the *Wolbachia* strain *w*AlbB annotated NCBI RefSeq assembly (GCF_004171285.1; Sinha et al., 2019), the *A. albopictus* host NCBI RefSeq assembly (GCF_035046485.1), which includes the host mitochondrial genome (NC_006817.1). In addition, to account for all potential sources of RNA transcripts in our samples, the genome sequences of various arthropod-specific viruses that have previously been reported to infect the Aa23 cell line (Hoshino et al., 2009; Bishop et al., 2020; Manni and Zdobnov, 2020; Parry et al., 2021) were also added to the composite reference file.

To ensure equivalent total starting read counts across all samples within a given experimental comparison, paired-end reads were randomly subsampled to match the library with the lowest sequencing depth. Specifically, samples were subsampled to 40 million reads for 1-round and 2-round enrichment comparisons, 19 million reads for Cappable-seq versus XRN-1 comparisons, and 29 million reads per replicate per day for the time-course differential expression analyses. The subsampled reads were then mapped to the combined host-endosymbiont-viral reference genomes index using the BWA-MEM algorithm from the BWA package (v0.7.18-r1243-dirty) (Li, 2013). Gene and gene biotype annotations for *w*AlbB were obtained from the GTF file in the NCBI RefSeq database. This GTF annotation was also provided to the featureCounts tool within the subread package (v2.1.1) (Liao et al., 2014) to generate normalized read counts (CPMs) for each feature using stranded library option (-s 2). The proportion of reads mapping to various gene biotypes within each species was quantified and plotted using custom R scripts.

Metatranscriptome assembly was performed for each treatment condition using rnaSPAdes (v4.1.0) (Bushmanova et al., 2019) in stranded mode (command-line option “–ss fr”). To search for potential RNA viruses, assembled transcripts originating from mosquito and *Wolbachia* were first identified and removed by aligning to the respective reference genomes using minimap2 (v2.28-r1209) (Li, 2018) with command-line options “-ax -asm5.” Within the remaining contigs, amino-acid sequences from all open reading frames were obtained using getORF utility from the EMBOSS suite (Rice et al., 2000) and used as input to the RdRp-scan software (v0.90) (Charon et al., 2022). Transcript contigs with a positive hit for RdRp domains were selected as potential RNA virus transcripts, and their species of origin was confirmed by analyzing their BLASTN hits (Camacho et al., 2009) against the NCBI Core nucleotide database (“core_nt,”^1^). Per-base depth of coverage for viral sequences was obtained using samtools (v 1.22.1) (Danecek et al., 2021) via the depth command with “-a” option to include positions with zero coverage. The assembled virus sequences were deposited to GenBank database under BioProject PRJNA1392518 and are also made available at the GitHub repository accompanying this manuscript.

Transcript quantification and differential expression analysis were conducted using kallisto (v 0.51.1) (Bray et al., 2016) and edgeR (v4.8.0) (Chen et al., 2025). Genes were classified as significantly differentially expressed (DEGs) based on a false discovery rate (FDR)-corrected *p*-value of <0.05. Heatmaps displaying the z-scores of normalized counts were generated using pheatmap package (v 1.0.13) in R. Finally, to functionally characterize the DEGs, eggNOG, and PFAM annotations from the original assembly (Sinha et al., 2019) were transferred over to latest RefSeq gene models. The commands, scripts, gtf format files for RefSeq gene models and other metadata used in the analyses are available at the “metadata” folder at https://github.com/aWormGuy/Wolbachia-Cappable-seq-Differential-Expression/.

## 3 Results

### 3.1 Cappable-seq enriches the active, non-rRNA transcripts from the *Wolbachia* strain *w*AlbB

**FIGURE 1:**
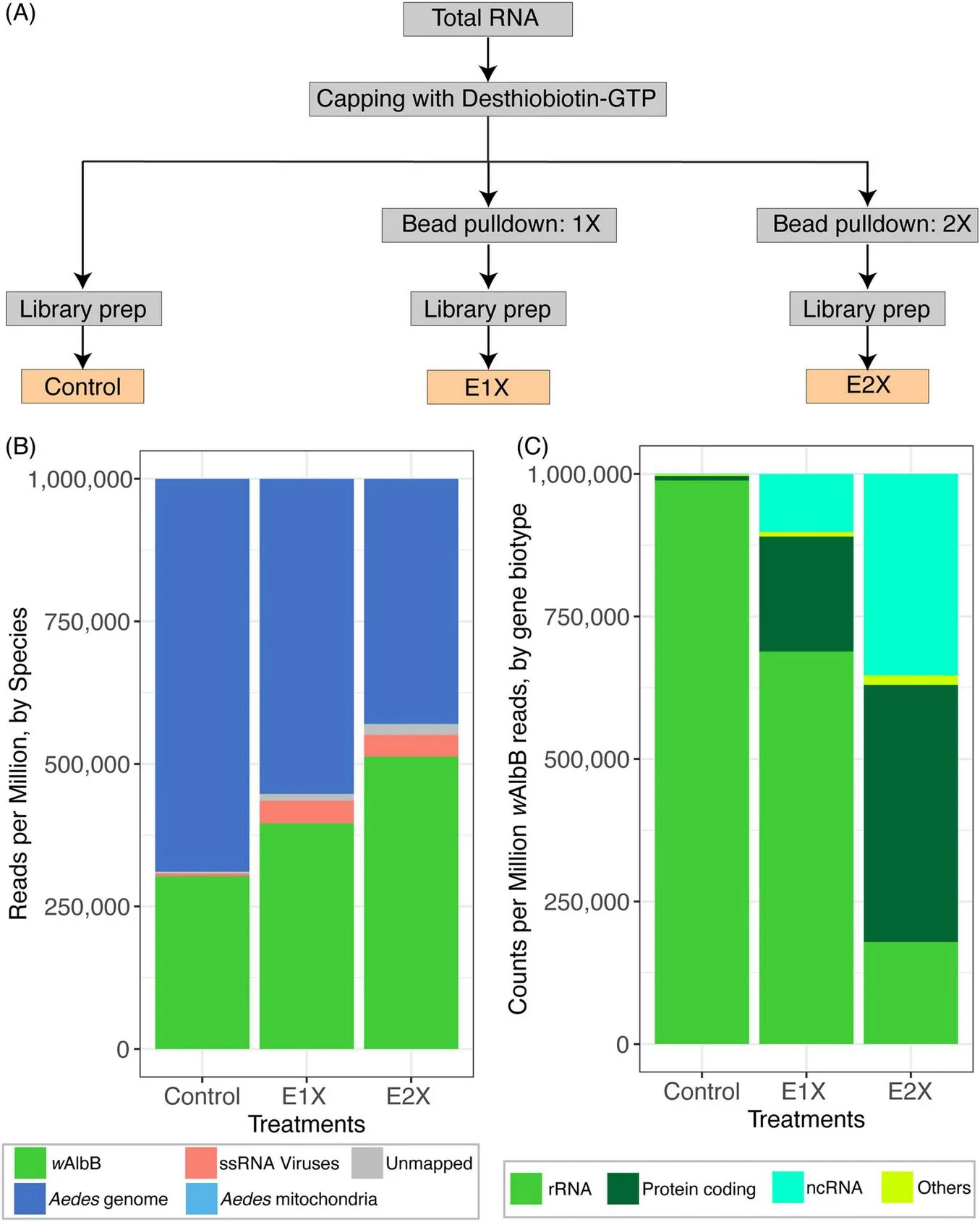
Cappable-seq enriches *Wolbachia* primary transcriptome. **(A)** Schematic of the experiment comparing efficiencies of Cappable-Seq based enrichment of *w*AlbB RNAs. **(B)** Distribution of total mapped sequencing reads, expressed as Reads Per Million (RPM), categorized by source genome across three library preparations: an un-enriched control, one round of beads enrichment (E1X), and a two-round enrichment (E2X). **(C)** Distribution of *Wolbachia* reads mapped to various gene features, expressed as Counts Per Million (CPM), categorized by gene biotype across the same library preparations.

To evaluate the efficacy of Cappable-seq in isolating endosymbiont transcripts from a complex intracellular environment, we extracted total RNA from Aa23 cells infected with *w*AlbB. We compared the distribution of mapped sequencing reads across three library preparations: an un-enriched control, a library subjected to a single round of streptavidin bead enrichment (1x), and a library subjected to two sequential rounds of enrichment (2x) (Figure 1A).

At the species level, the un-enriched control library was dominated by host transcripts, with 70% of the reads mapping to the mosquito genome while the remaining ∼30% mapped to the *w*AlbB genome (Figure 1B). Upon a single round of streptavidin bead enrichment, the *w*AlbB reads increased to roughly 40% of the library. A second sequential round of enrichment further elevated *w*AlbB reads to over 50% of the total library.

Beyond inter-species read distribution, the utility of a bacterial transcriptomic library is ultimately defined by its coverage of actively transcribed messenger RNA (mRNA) in the target bacterial species. Analysis of gene biotypes within the total *Wolbachia* reads established that the un-enriched *Wolbachia* transcriptional profile was overwhelmingly dominated by rRNA (∼98%), with only a negligible fraction of reads mapped to protein-coding regions (Figure 1C). The first and second rounds of Cappable-seq enrichment reduced the *w*AlbB rRNA fraction down to ∼68% and 18%, respectively, while the protein-coding transcripts expanded to nearly 20% and 45% of the library, respectively (Figure 1C). These key enrichment outcomes can also be visualized in a genome-browser view (Supplementary Figure 2), where the genome-wide coverage tracks show sparse coverage in the un-enriched control, insufficient to resolve full gene architectures. In contrast, single-round and double-round Cappable-seq yielded deep, continuous, and extensive coverage across the entire transcript regions of non-rRNA genes (Supplementary Figure 2). Based on these results, a capping reaction followed by two rounds of bead-based enrichment was chosen as the standard protocol for the preparation of subsequent Cappable-seq libraries.

### 3.2 Cappable-seq enhances the whole-genome coverage of multiple ssRNA viruses

**FIGURE 2:**
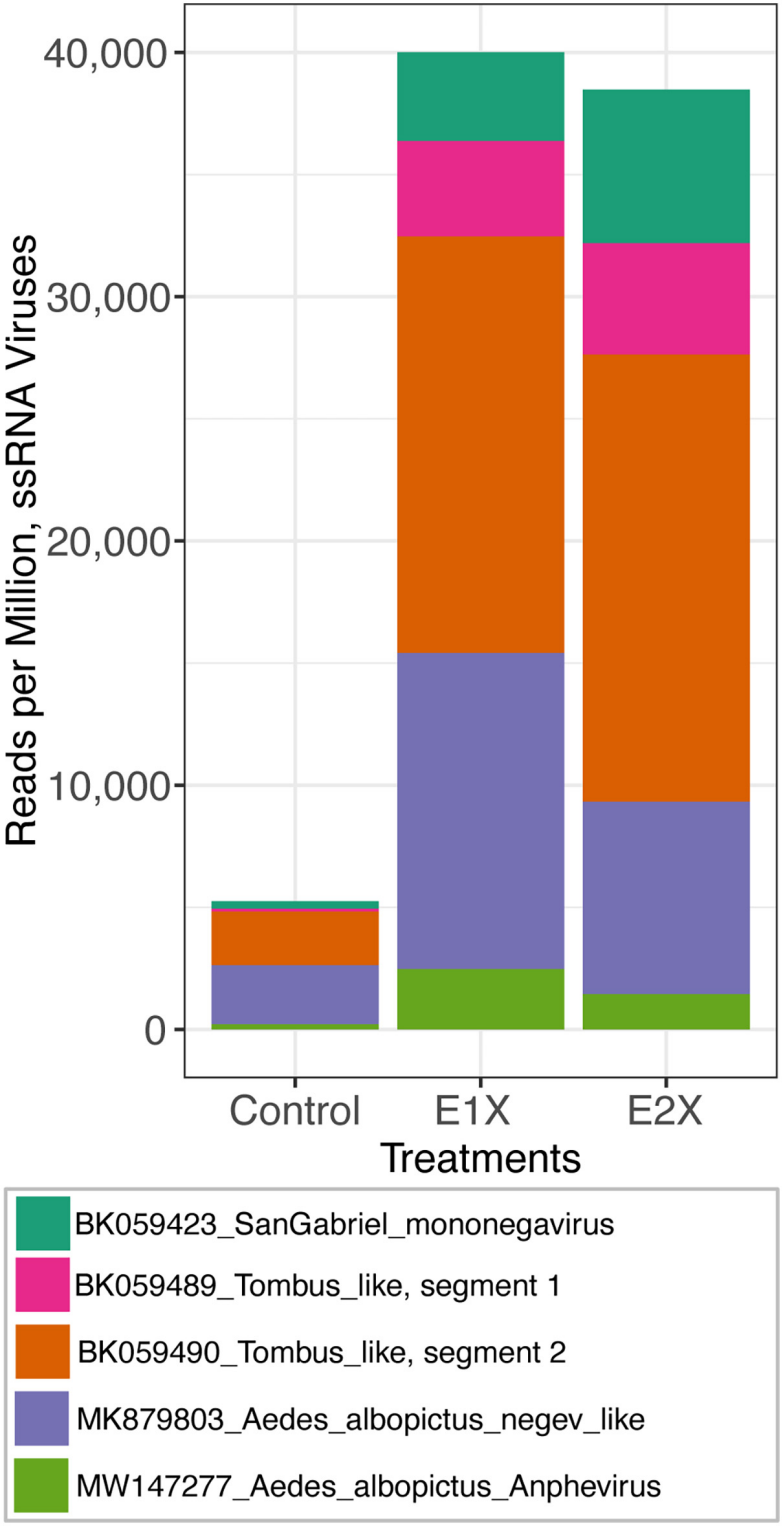
Cappable-seq captures the active endogenous viral community within the *Aedes albopictus* Aa23 mosquito cell line. Absolute abundance (RPM) and species-level breakdown of mapped viral reads across the un-enriched and enriched libraries.

Multiple arthropod-specific viruses have previously been reported in the Aa23 cell line (Hoshino et al., 2009; Bishop et al., 2020; Manni and Zdobnov, 2020; Parry et al., 2021). In addition to the bacterial transcripts from *w*AlbB, the Cappable-seq libraries also revealed a robust co-enrichment of reads originating from these single-stranded RNA (ssRNA) viruses. While viral reads were nearly undetectable in the standard control library, they were distinctly captured and progressively enriched across the 1x and 2x libraries (Figure 1B). This captured metavirome consists of two positive single-strand RNA viruses (++ssRNA), namely the *A. albopictus* negev-like virus, and the Tiger mosquito bi-segmented tombus-like virus, as well as two negative single-strand RNA (−ssRNA) viruses, namely the *A. albopictus* anphevirus and the San Gabriel mononegavirus (Figure 2). The enrichment in the number of reads after Cappable-seq also contributed to an increase in depth of coverage across the entire genomes, with the most dramatic increases observed at the 5-prime ends of viral genes (Supplementary Figures 3A–E).

To further investigate the utility of Cappable-seq for reference-free discovery of ssRNA viruses, an unbiased, *de novo* assembly of RNA-seq reads was also performed for each treatment, and potential viral contigs were identified using RdRp-scan. For each of the four viruses described above, the corresponding full-length assembled sequences were successfully recovered, with higher depth of coverages observed in Cappable-seq libraries relative to the control library.

In addition, two new potential viral contigs was identified from the RdRp-scan results. An assembled fragment of 2,967 nt showed 98% identity over its entire length to a 3,016 nt sequence from a negative-stranded ssRNA Solemoviridae virus isolate (accession PQ683188.1) from meta-transcriptome study of mosquito larvae from Georgia, United States. Another contig with size of 2,487 nt showed 84% identity across 95% of its length to a 2,712 nt sequence from an *Aedes* birnavirus (accession MT381948.1), a double-stranded RNA virus reported from meta-transcriptome studies of mosquito isolates from Australia (O’Brien et al., 2020). Interestingly, for the Solemoviridae-like contig, practically no enrichment was observed in the E1X experiment, and a depletion was observed in the E2X experiment (Supplementary Figure 4A), while a modest 1.5X and increase in the coverage in Cappable-seq libraries was observed for the *Aedes* Birnavirus-like contig (Supplementary Figure 4B).

Together, these data demonstrate that in addition to enriching and capturing the active primary transcriptomes of bacterial endosymbionts, the Cappable-seq protocol effectively enriches the genomes and transcripts from co-infecting ssRNA viruses and could be a promising novel tool for *de novo* metaviromics and genome assembly.

### 3.3 Cappable-seq outperforms XRN-1 exonuclease treatment for *Wolbachia* mRNA enrichment

**FIGURE 3:**
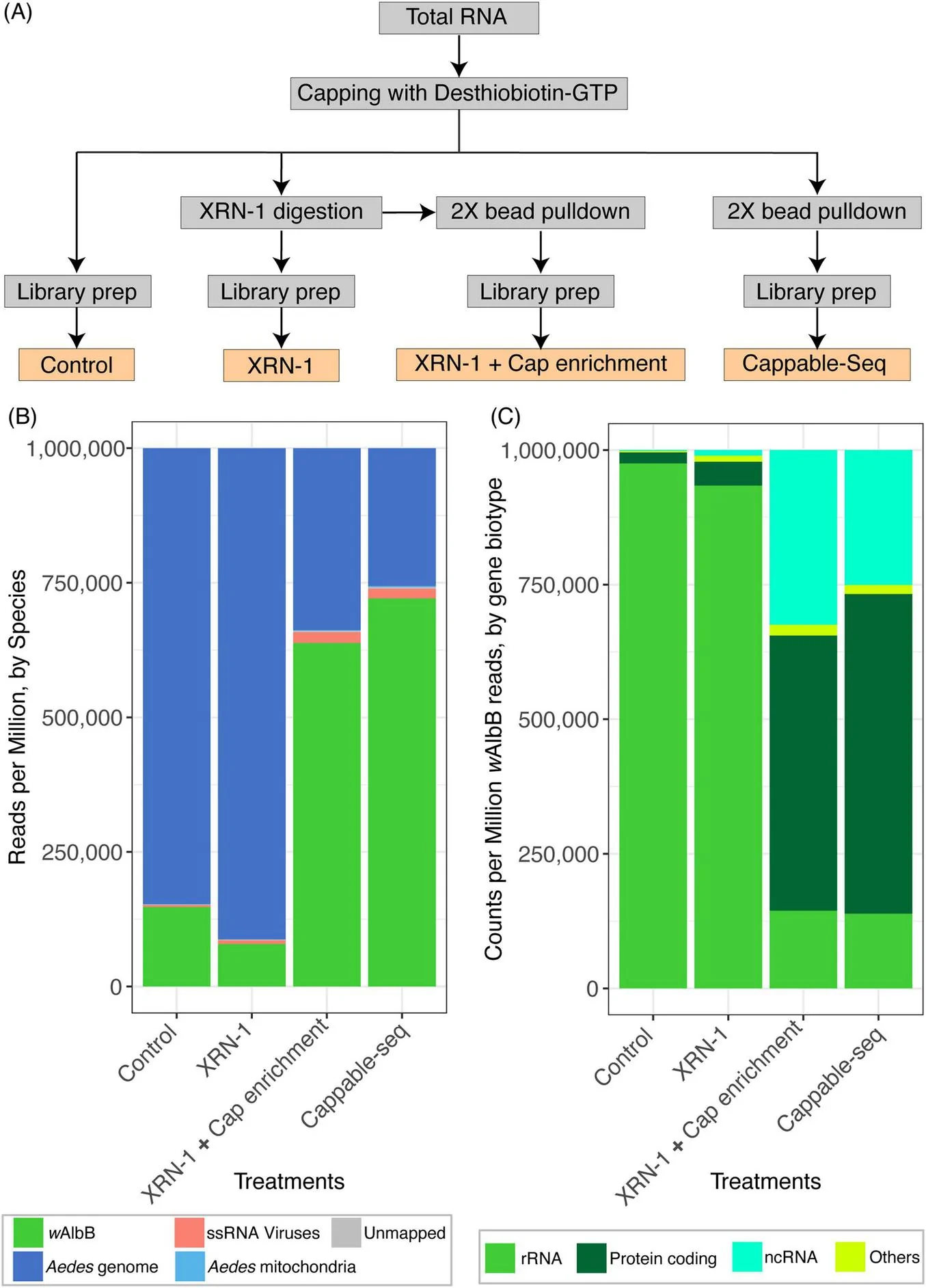
Target enrichment via Cappable-seq outperforms alternative degradation methodologies. **(A)** Schematic of the experiment comparing efficiencies of Cappable-Seq based enrichment of *w*AlbB RNAs to alternative treatments. **(B)** Species-level distribution of total mapped sequencing reads (RPM) categorized by source genome, comparing un-enriched controls libraries to those treated with XRN-1 exonuclease alone, XRN-1 followed by bead-based enrichment of Capped RNAs, and Cappable-seq alone. **(C)** Distribution of mapped *Wolbachia* reads (CPM) categorized by gene biotype across the respective treatments.

A common alternative strategy for enriching prokaryotic transcripts in mixed samples relies on degradation rather than physical capture. XRN-1 is a highly active 5′-to-3’ exoribonuclease that specifically degrades RNAs with 5′-monophosphate ends, characteristic of processed rRNA and many degraded transcripts, while leaving the prokaryotic primary transcripts, which have a 5′-triphosphate, and mature eukaryotic transcripts, which have a 5′-capped end intact. To compare Cappable-seq versus the XRN-1 mediated degradation approach for *w*AlbB mRNA enrichment, we prepared four RNA libraries with different treatments after the capping reaction (i) an un-enriched control, without any bead-based enrichment, (ii) XRN-1 alone, without any bead-based enrichment (iii) XRN-1 treatment plus bead-based enrichment, and (iv) standard Cappable-seq workflow via bead-based enrichment (Figure 3A).

On a sample containing total RNA from both the host and symbiont, using Cappable-seq protocol alone yielded the highest enrichment, increasing the *w*AlbB proportion to over 72% and robustly capturing the ssRNA viral transcripts as well (Figure 3B). The XRN-1 treatment alone not only failed to enrich the *Wolbachia* transcriptome, but it also slightly decreased the fraction of reads mapped to *Wolbachia* from 14 % in the control library to about 8 % in the XRN-1 library. One reason for this result is that even though XRN-1 would have digested the host rRNA, the eukaryotic host mRNA is inherently protected by a 5′-cap from XRN-1 digestion, thereby contributing to greater fraction of mosquito reads. Notably, pre-treating the RNA with XRN-1 prior to Cappable-seq enrichment offered no additional *w*AlbB enrichment as compared to the Cappable-seq alone treatment (Figure 3B).

We observed a similar trend when analyzing the ability of these treatments to enrich the *w*AlbB mRNA compared to other gene biotypes such as the rRNA. In the un-enriched control, rRNA constituted over 95% of the *w*AlbB reads. Cappable-seq treatment successfully decreased the *w*AlbB rRNA fraction to under 15% and increased the protein-coding mRNA to about 60% of all the *Wolbachia* mapped reads. The ncRNA fraction also increased to 25% in the Cappable-seq library. In contrast, treatment with XRN-1 alone only marginally reduced rRNA proportion to ∼90%, providing very little gain for the protein-coding fraction (Figure 3C). Since XRN-1 specifically targets processed rRNAs, we evaluated whether pre-treating the sample with XRN-1 could synergize with Cappable-seq to further suppress the remaining ribosomal transcripts observed in the Cappable-seq alone treatment. However, this combination offered no additional ribosomal depletion and yielded a slightly lower protein-coding fraction (∼50%) compared to the standalone Cappable-seq protocol.

Viral read abundance also followed a similar pattern. Briefly, Cappable-seq robustly captured and enriched the ssRNA viral community, heavily enriching the relative abundance of all four viral species. XRN-1 treatment alone had a negligible effect compared to the un-enriched control. Interestingly, while the combination of XRN-1 pre-treatment followed by Cappable-seq yielded the highest overall viral reads per million (RPM) of *A. albopictus* negev-like virus sequence MK879803 (Supplementary Figure 5), the standalone Cappable-seq protocol still provided robust viral capture.

Collectively, these results demonstrate that Cappable-seq is superior to XRN-1 exonuclease digestion for host background depletion and bacterial rRNA removal. Furthermore, the robust efficiency of the capping enzyme and streptavidin enrichment makes prior XRN-1 digestion unnecessary, establishing Cappable-seq as a highly efficient, standalone methodology for studying intracellular transcriptomics.

### 3.4 *Wolbachia* intracellular density increases steadily during a standard host-cell passage

**FIGURE 4:**
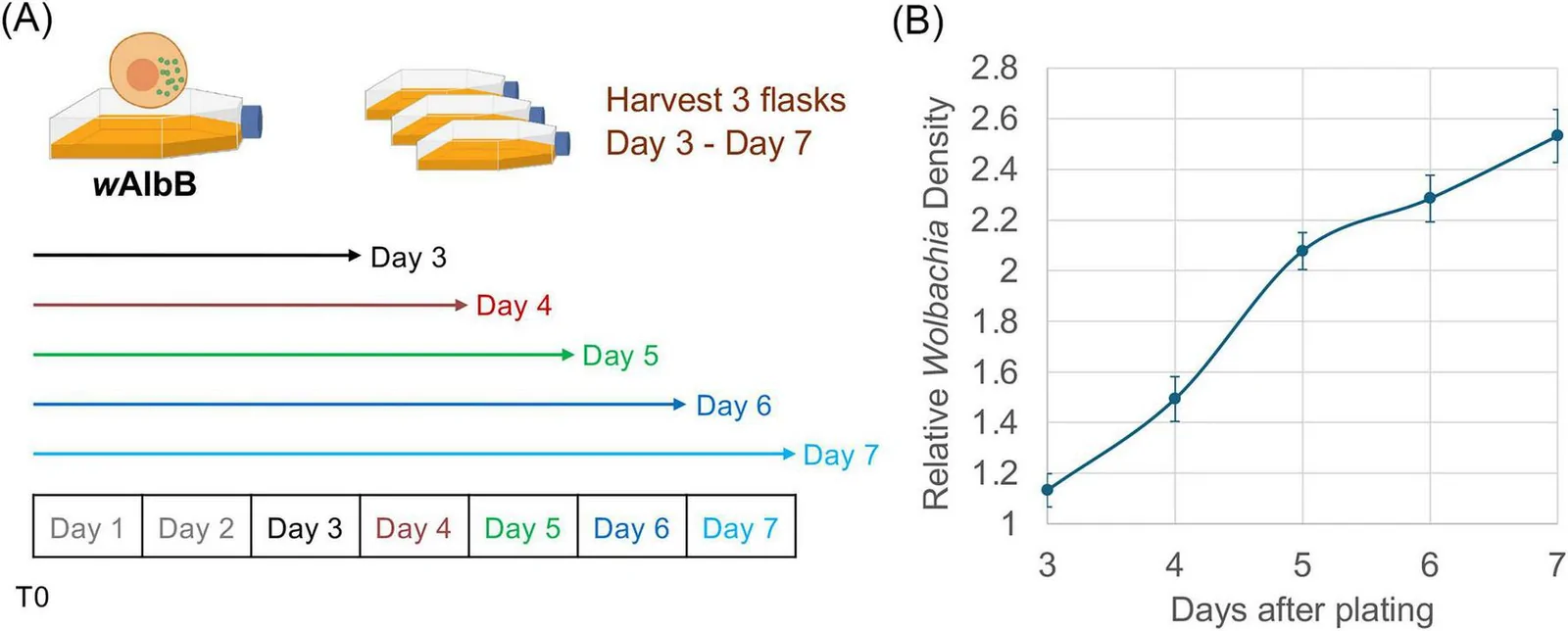
Temporal dynamics and intracellular expansion of *Wolbachia* during one host cell passage. **(A)** Schematic representation of the 5-day time-course sampling strategy in the *A. albopictus* Aa23_*w*AlbB cell line, spanning early exponential growth (Day 3) to late confluency (Day 7). **(B)** Relative intracellular *Wolbachia* density across the 5-day passage, quantified via TaqMan multiplex qPCR. Density was calculated using the relative standard curve method and is expressed as the normalized ratio of the bacterial 16S rRNA target quantity to the host mosquito 18S rRNA target quantity. Data represent the mean of three independent biological replicates; error bars indicate standard error of the mean (SEM).

Having established Cappable-seq as a robust methodology for isolating endosymbiont mRNA, we applied this approach to investigate the transcriptional dynamics of *Wolbachia* during an active cellular infection time course using the Aa23_*w*AlbB cell line (Figure 4A). Following passage into fresh medium, triplicate flasks were harvested daily starting from Day 3 (early exponential growth) through Day 7 (late confluency) (Supplementary Figure 6). We monitored the *Wolbachia* growth kinetics using quantitative PCR (qPCR) to determine the relative abundance of bacterial 16S and host mosquito 18S rRNA gene copies from genomic DNA extracted at each time point. The relative *Wolbachia* density exhibited a continuous, steady increase as the host cell population expanded, effectively doubling in relative titer from Day 3 to Day 7 (Figure 4B). This confirmed that our sampling window successfully captured an active phase of intracellular bacterial replication and expansion, providing a physiologically relevant model to study density-dependent shifts in *Wolbachia* gene expression.

### 3.5 Global transcriptomic profiling reveals a coordinated transcriptional regulation in *Wolbachia* during a culture time-course

To capture the transcriptomic dynamics of the intracellular expansion of *Wolbachia*, total RNA from the Day 3 through Day 7 biological triplicates was subjected to the optimized two-round Cappable-seq protocol. Analysis confirmed highly consistent transcript capture, with *w*AlbB reads stably comprising approximately 80% of the total sequenced reads and the co-enriched viral community maintaining a stable abundance throughout the culture period (Supplementary Figure 7).

**FIGURE 5:**
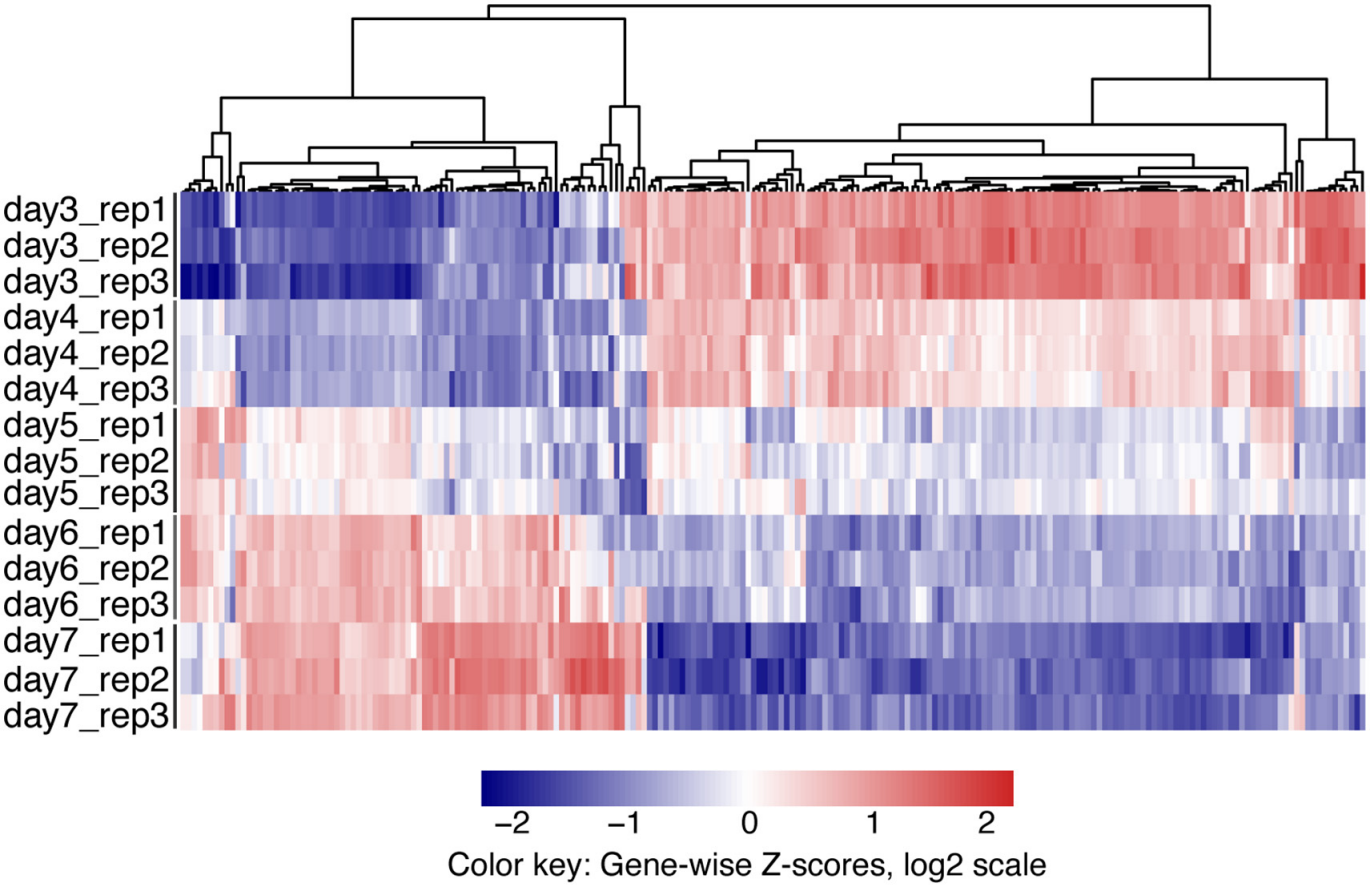
Density-dependent global transcriptional reprogramming of *Wolbachia*. Heatmap detailing the relative expression profiles of all 216 significantly differentially expressed genes across the 5-day culture time-course. Rows represent individual biological replicates per time point, and columns represent individual genes.

Distinct and reproducible transcriptional profiles were observed for each sampled time-point. A multi-dimensional scaling plot based on normalized *w*AlbB gene counts showed biological replicates for each sample clustering together while remaining distinct from clusters corresponding to other time points (Supplementary Figure 8A). Applying a differential expression threshold of FDR < 0.05 and an absolute fold-change ≥ 1.4 relative to Day 3, a total of 216 *w*AlbB genes were identified as being differentially regulated during the culture time-course (Supplementary Table 1). The number of both upregulated and downregulated genes expanded steadily across the time course, ranging from 6 to 75 and 33 to 133 genes, respectively (Supplementary Figure 8B). This coordinated temporal shift is also captured in the global gene expression heatmap (Figure 5).

To define the specific biological pathways driving this density-dependent shift, we focused on genes exhibiting continuous, unidirectional regulation across the time points and found that 102 genes were continuously downregulated from Day 4 through Day 7, compared to only 30 continuously upregulated genes. The continuously upregulated set was enriched for genes involved in translation (elongation factor Tu, locus DEJ70_RS01770; tmRNA gene ssrA, locus DEJ70_RS04165), metabolism (DEJ70_RS03670, glpX fructose-bisphosphatas, SemiSWEET family sugar transporter, pyridoxine 5′-phosphate synthase pdxJ, DEJ70_RS01400) and stress response genes such as the RNA polymerase factor sigma-32 rpoH gene (locus DEJ70_RS02185), and the Hsp20/alpha crystallin family protein encoded by hspC2 (locus DEJ70_RS01395). A distinct Ankyrin repeat-containing gene (DEJ70_RS04245) was continuously upregulated, as was an HK97 family phage prohead protease gene (DEJ70_RS03385) indicating the activation of stress-induced response as the culture reached late confluency. Interestingly the non-coding 6S RNA gene ssrS (DEJ70_RS02130) which functions as a global transcriptional regulator, was also showed continuously increasing fold-changes during the culture time course, suggesting its important role in *Wolbachia* transcriptional regulation.

The set of continuously downregulated genes was enriched for proteins containing eukaryotic-like protein-protein interaction modules. This included six different Ankyrin-repeat domain containing proteins (ANKs) (DEJ70_RS01890, DEJ70_RS04425, DEJ70_RS05810, DEJ70_RS01950, DEJ70_RS05400, DEJ70_RS02825), three coiled-coil domain containing proteins (DEJ70_RS00020, DEJ70_RS01990, DEJ70_RS00655), and a tetratricopeptide repeat protein (DEJ70_RS07105), which are widely utilized to mediate interactions with the eukaryotic host. In addition, multiple P44/Msp2 family outer membrane proteins and components of Type IV secretion systems were also downregulated (Supplementary Table 1). This targeted suppression of the host interface coincided with a broad downregulation of basic energetic transporters, including MFS transporters, ABC transporter binding proteins, and cation/proton antiporters.

## 4 Discussion

Defining the active transcriptome of obligate intracellular bacteria is fundamentally hindered by the overwhelming abundance of eukaryotic host RNA, which routinely masks the bacterial transcriptional profile in standard sequencing approaches. In this study, we demonstrate that Cappable-seq provides a robust, targeted solution to this barrier by specifically capturing and enriching the 5′-triphosphate primary transcripts from *Wolbachia*. This methodology not only significantly reduced the *A. albopictus* host background but also functioned as a highly efficient bacterial rRNA reduction method, outperforming the commonly used XRN-1 exonuclease treatments.

Although Cappable-seq was primarily deployed here to isolate the *Wolbachia* endosymbiont transcriptome, the protocol simultaneously revealed the robust co-enrichment of a stable, multi-species viral community within the *A. albopictus* Aa23 cell line. The presence of arthropod-specific viruses is a well-documented phenomenon in mosquito-derived cell lines (Hoshino et al., 2009; Bishop et al., 2020; Manni and Zdobnov, 2020; Parry et al., 2021). While no reads were found mapped to *Aedes* flavivirus, we identified a diverse viral community comprising both +ssRNA and −ssRNA viruses, including *A. albopictus* negev-like virus, *A. albopictus* anphevirus, San Gabriel mononegavirus, and a bi-segmented tombus-like virus. During ssRNA virus transcription, the mature transcripts are typically capped co-transcriptionally. However, the enrichment of these viral transcripts via Cappable-seq underscores important aspects of viral RNA biology that warrant further study. For viral families that canonically cap their mature transcripts, it appears that the abundance of nascent replicative intermediates and anti-genomes possessing transient 5′-triphosphate ends during the intracellular viral replication enables their capture by the Cappable-seq protocol. In addition, because the members of the *Tombusviridae* family natively lack a 5′ cap (Sit and Lommel, 2015), their genomes can serve as direct substrates for the capping enzyme used in this protocol. Therefore, Cappable-seq could also serve as an efficient tool for profiling active viral replication. Given that *Wolbachia* is known to modulate and inhibit certain insect-specific viruses, such as the *A. albopictus* negev-like virus, the ability of Cappable-seq method to simultaneously resolve both the endosymbiont and the actively replicating and transcribing virome provides a uniquely comprehensive view of the intracellular environment.

In addition, we demonstrate the utility of Cappable-seq for the *de novo* assembly and discovery of ssRNA viruses, facilitated by the high enrichment of reads originating from ssRNA viruses. Full-length viral genome segments containing RdRp domains were successfully assembled for all four ssRNA viruses known to be present in this cell line. Intriguingly, two new potential viral sequences assembled from our data, namely a Solmoviridae-like ssRNA virus contig and a dsRNA *Aedes* birnavirus-like contig showed an exception to this enrichment trend. This exception implies that these viruses either have differences in their transcription and replication biology and in their 5′-end structures, or they are present as endogenous virus elements integrated within the *A. albopictus* mosquito genome with eukaryotic transcript features (Palatini et al., 2022).

By applying this high-resolution transcriptomic approach to track the *Wolbachia w*AlbB strain across a host cell passage, we observed a density-dependent global transcriptional shift. We observe the activation and continuous upregulation of specific stationary-phase and stress-response factors of the Hsp20/alpha crystallin family and the sigma-factor *rpoH*, indicating a robust response to the physiological stress of intracellular crowding. The concurrent transcriptional activation of HK97-family WO prophage elements implies that this high-density state may serve as a trigger for localized prophage activation. Together, these transcriptomic dynamics illustrate that *Wolbachia* is not static within the mosquito cell line. Instead, it actively senses its environment and balance host colonization with long-term survival within the dynamic constraints of its host cells. Having demonstrated the ability to analyze *Wolbachia* transcriptome at high resolution with this transcriptomic approach, we believe that it will enable further transcriptomic studies of host-symbiont interactions under various conditions and treatments.

While the Aa23 cell line provides a well-controlled *in vitro* environment to model density-dependent growth and study temporal transcriptomic variables, it inherently cannot capture the full physiological complexity of an intact arthropod host. Within the mosquito vector, *Wolbachia* exhibits pronounced tissue tropism (Dobson et al., 1999), colonizing tissues such as the midgut, hemolymph, and reproductive organs, each representing a distinct metabolic landscapes. How *Wolbachia* spatially and temporally modulates its transcriptome within these distinct *in vivo* niches remains a critical question. Cappable-seq is well-suited for mapping tissue-specific *Wolbachia* expression profiles and resolving the active, tripartite transcriptional interplay between the endosymbiont, the mosquito host, and co-infecting viruses. A foreseeable challenge in such experiment could be the relatively low-titers of *Wolbachia* in specific tissues or conditions where the relative titer of *Wolbachia* to host is lower than the cell-line used in the current manuscript. In principle, since Cappable-seq is a positive enrichment strategy where bacterial transcripts are enriched away from undesired host RNA, the method is expected to be successful in such cases. Indeed, a previous study successfully used Cappable-seq to enrich transcripts from a low-titer *Wolbachia w*MelPop-CLA infecting another *A. albopictus* cell-line, RML-12, as well as from low-titer *w*AlbB in Aa23 cells following antibiotic and heat-shock treatments (Suliman et al., 2025). The application of Cappable-seq to other host-microbiome systems remains to be tested in future studies.

In conclusion, resolving the dynamic transcriptomic profiles of obligate intracellular bacteria requires methodologies that can efficiently bypass the overwhelming transcriptional noise of the eukaryotic host. Our application of Cappable-seq to the *Wolbachia*-host system demonstrates a robust, 5′-triphosphate-directed enrichment strategy that successfully captures the active endosymbiont transcriptome while simultaneously depleting the structural rRNA population. By deploying this targeted tool across an active cellular infection cycle, we provide high-resolution evidence that *Wolbachia* does not maintain a static intracellular presence, rather, it executes a density-dependent global reprogramming for long-term intracellular persistence. As the field looks toward new frontiers in *Wolbachia* biology, overcoming these technical barriers is essential. This methodology, along with the foundational temporal dataset presented here, provides a critical framework for future investigations into how this masterful endosymbiont continuously senses, adapts to, and governs its complex intracellular niche.

## Data Availability

The datasets presented in this study can be found in online repositories. Raw sequence data and assembled virus sequences can be found in the NCBI database under the BioProject accession number PRJNA1392518.

## Footnotes

1
https://blast.ncbi.nlm.nih.gov/Blast.cgi
