# Supplementary material for: Primary transcript enrichment reveals a density-dependent Wolbachia transcriptome and active mosquito RNA viruses
Source: Front Microbiol. 2026 Aug 12;17:1860204. doi: 10.3389/fmicb.2026.1860204 (PMC13506747; doi:10.3389/fmicb.2026.1860204)
Supplement: Supplementary file 1 [file Data_Sheet_1.docx]

Supplementary Material

# 1 Supplementary Figures and Tables

**Supplementary Figure 1. Multiplex qPCR amplification and standard curves for relative quantification of *Wolbachia* density.** **(A)** Amplification plot (Relative Fluorescence Units vs. Cycles) of the 7-point, 10-fold serial dilution series for the *w*AlbB 16S rRNA gene target (FAM channel). **(B)** Standard curve generated from the 16S rRNA amplification data demonstrating high linearity and efficiency (Efficiency = 98.9%, R² = 0.999, Slope = -3.347). **(C)** Amplification plot of the corresponding 7-step, 10-fold serial dilution series for the *Aedes albopictus* Aa23 18S rRNA host gene target (HEX channel). **(D)** Standard curve generated from the 18S rRNA amplification data (Efficiency = 100.2%, R² = 0.999, Slope = -3.317). All standard curves were run in technical triplicates.

**Supplementary Figure 2. A representative Integrated Genomics Viewer (IGV) view of mapped reads and read coverage across a ~10 kb genomic locus (NZ_CP031221.1:747,907-758,588) on the *w*AlbB chromosome.** Data tracks for un-enriched control (top), 1-round (Enrich_1x), and 2-round (Enrich_2x) library preparations show read coverage depth (gray area) from 40 million mapped reads, plotted on a log10 scale. Individual mapped reads are colored red or blue to indicate strand direction. Peak coverage depth values for each track are Control [0 - 15], Enrich_1x [0 - 916], and enrich_2x [0 - 2014]. Gene models at the bottom indicate the 11 genes within the locus.

**Supplementary Figure 3. Integrated Genomics Viewer (IGV) view showing effect of Cappable-Seq treatment on the number of mapped reads and coverage depth across 4 ssRNA viruses**. (A) *Aedes albopictus* anphevirus (B) San Gabriel mononegavirus (C) *Aedes albopictus* negev-like virus (D) Tiger mosquito bi-segmented tombus-like virus, segment 1, and (E) Tiger mosquito bi-segmented tombus-like virus, segment 2.

**Supplementary Figure 4. Integrated Genomics Viewer (IGV) view showing effect of Cappable-Seq treatment on the number of mapped reads and coverage depth across the *de novo* assembled, potential viral contigs.** (A) Solemoviridae-like contig (B) Aedes Birnavirus-like contig.

**Supplementary Figure 5.** Absolute abundance (RPM) and species-level breakdown of mapped viral reads across the un-enriched control and alternative enrichment/degradation library preparations.

**Supplementary Figure 6.** Representative bright-field micrograph of the Aa23 cell line across the culture passage including the images at the initiation of the time course (Day 0, 4 hours post-plating) and subsequently on Days 3, 4, 5, 6, and 7**.**

**Supplemental Figure 7. Longitudinal stability of Cappable-seq capture efficiency across a 5-day culture passage.** **(A)** Mean distribution of total mapped sequencing reads (RPM) by source species for enriched libraries across the Day 3 through Day 7 time-course. **(B)** Mean biotype distribution (CPM) of captured *Wolbachia* transcripts across the same temporal window. **(C)** Mean absolute abundance (RPM) of the co-enriched multi-species viral community throughout the time-course. Data represent the mean of three independent biological replicates per time point.

**Supplementary Figure 8. Density-dependent global transcriptional reprogramming of *Wolbachia*.** (A) Multi-Dimensional Scale (MDS) plot of *Wolbachia* global transcriptomes from Day 3 through Day 7. (B) Total number of differentially expressed *Wolbachia* genes (DEGs) across the culture passage, relative to the Day 3 early-infection baseline. Bars represent the absolute number of significantly upregulated (red) and downregulated (blue) genes at each respective time point.

**Supplementary Table 1.** Complete list of differentially expressed *Wolbachia w*AlbB genes and functional annotations across the host-cell passage time course.
